# The Effects of Cranial Orientation on Forensic Frontal Sinus Identification as Assessed by Outline Analyses

**DOI:** 10.3390/biology11010062

**Published:** 2022-01-02

**Authors:** Lauren N. Butaric, Allison Richman, Heather M. Garvin

**Affiliations:** Department of Anatomy, Des Moines University, 3200 Grand Avenue, Des Moines, IA 50312, USA; Allison.Richman@dmu.edu (A.R.); Heather.Garvin-Elling@dmu.edu (H.M.G.)

**Keywords:** human identification, frontal sinus shape, outline analysis, elliptical Fourier analysis, computed tomography, radiology

## Abstract

**Simple Summary:**

Frontal sinus patterns are unique amongst individuals. When faced with an unknown decedent, investigators can compare the frontal sinus pattern observed in postmortem radiographs to antemortem radiographs of the suspected individual to make a positive identification. Ideally, the antemortem and postmortem radiographs are oriented in the same exact position, but this can be challenging. This study investigates how slight variations in radiographic orientation affect sinus outlines and potentially impact identification. Frontal sinus models were created from CT scans (21 individuals) and digitally oriented across three clinically relevant views. From each standard orientation (looking straight ahead), eight 5° deviations were obtained in horizontal (left/right), vertical (up/down), and diagonal (e.g., left-up vs. right-down) directions. Within and between individual differences in sinus size and outline shape were assessed. Sinus breadth remained relatively stable across deviations, while sinus height was affected by small vertical deviations. Although radiographic vertical deviations resulted in statistical differences, impacts on outline matches were minimal. However, practitioners need to take particular care in matching radiographic orientation for smaller and/or discontinuous (right and left sides separated) sinuses, which are more likely to lose part of the sinus in more inferiorly oriented views and, thus, could affect various methods of sinus identification.

**Abstract:**

The utility of frontal sinuses for personal identification is widely recognized, but potential factors affecting its reliability remain uncertain. Deviations in cranial position between antemortem and postmortem radiographs may affect sinus appearance. This study investigates how slight deviations in orientations affect sinus size and outline shape and potentially impact identification. Frontal sinus models were created from CT scans of 21 individuals and digitally oriented to represent three clinically relevant radiographic views. From each standard view, model orientations were deviated at 5° intervals in horizontal, vertical, and diagonal (e.g., left-up) directions (27 orientations per individual). For each orientation, sinus dimensions were obtained, and outline shape was assessed by elliptical Fourier analyses and principal component (PC) analyses. Wilcoxon sign rank tests indicated that sinus breadth remained relatively stable (*p* > 0.05), while sinus height was significantly affected with vertical deviations (*p* < 0.006). Mann–Whitney U tests on Euclidean distances from the PC scores indicated consistently lower intra- versus inter-individual distances (*p* < 0.05). Two of the three orientations maintained perfect (100%) outline identification matches, while the third had a 98% match rate. Smaller and/or discontinuous sinuses were most problematic, and although match rates are high, practitioners should be aware of possible alterations in sinus variables when conducting frontal sinus identifications.

## 1. Introduction

The potential of radiographic comparisons and forensic identification based on frontal sinus morphology, in particular, has been recognized since the 1920s [1,2,3,4,5,6,7,8,9,10,11,12,13,14,15,16,17,18]. Frontal sinus morphology is highly individualized with differences noted even between monozygotic twins, which makes them an ideal candidate for identification [2,3,4,19]. If antemortem radiographic images containing the frontal sinus are available for a suspected decedent, a comparison with postmortem radiographs can provide a fast and inexpensive method of identification, similar to radiographic dental comparisons. Comparisons can also frequently be made on fragmented or even burned remains. Specific methods for radiographic frontal sinus identification range from qualitative visual assessments [1,3,19,20] to the use of metrics and/or coded traits [7,8,17,21,22], and outline analyses [15,16,23]—all of which rely at least partly on sinus shape as defined by the presence of right/left sinus lobes, as well as the individual scallops and arcades that give the sinus their distinctive outlines. 

All frontal sinus radiographic methods also require a postmortem radiograph taken in the same orientation as the antemortem record. Antemortem radiographs reflect standard clinical views typically used in sinus or head-and-neck imaging, with three common orientations being Caldwell’s, posterior–anterior (PA)-frontal, and Water’s view [24]. Forensic anthropologists are more familiar with the Frankfort Horizontal orientation of the cranium and may be inclined to take postmortem radiographs in this anthropological orientation; further, several studies on frontal sinus variation utilize this orientation [25,26]. These orientations vary in the positioning of the head/cranium relative to the film and the trajectory of the X-rays. As such, the appearance of the frontal sinus on the two-dimensional radiographs may be altered based on the radiographic orientation chosen. 

Given the potential effects of orientation on radiographic representation, practitioners acquiring postmortem radiographs should aim to position the cranium in a similar orientation as the antemortem view. Still, obtaining a perfect alignment, however, can be challenging. Owing to this, several previous studies have investigated how slight variations in skull orientation affect the radiographic presentations of frontal sinus morphology and individual identification methods [25,26,27,28,29]. Overall, studies suggest that even 5° degrees of varying orientation may affect sinus morphology. However, these previous studies are limited in scope. For example, when testing their outline-based method, Christensen [28] was only able to effectively measure two crania in a single clinical view. Silva et al. [25] investigated how varying 10° vertical orientations affects frontal sinus breadth, but only incorporated a single individual and limited analyses to PA-frontal view. It is possible that the degree of error introduced is dependent on sinus size and complexity. So, the degree of shape deviation obtained from these limited studies may depend on the frontal sinus morphology of the single individual used in the error analysis. 

In each of the above studies, the physical placement and repositioning of the crania was done by the technicians and could add human error/bias given the challenges of obtaining perfect alignments. In fact, Hashim et al. [29] found that repositioning of crania on radiographic tables, even after only a short time has passed between repositioning (less than one minute) results in significantly different sinus presentations. To account for this, Riepert et al. [27] utilized a specialized program that simulates radiographs from CT-derived data. These authors digitally re-oriented crania in 4° and 8° variations. Ultimately, they found that frontal sinus breadth and height presented with high degree of variability across the orientations, but the inter-individual uniqueness of the sinus was such that these variations did not result in misidentifications. More recently, Nikolova et al. [26] utilized an industrial µCT scanner to obtain radiographic images, which allowed re-orientation of crania using more precise means via a computer-automated tilting gantry. They compared linear measures of the frontal sinus across 10 orientations at 5° intervals, starting from the Frankfort Horizontal plane at −20° to a view at 45° with the midpoint being 0°. Overall, they found significant differences in height and breadth measures at 5° vertical variations from the 0° midpoint. While the use of an industrial scanner allowed hands-free vertical tilting of the cranium, they were unable to incorporate lateral movements. Further, they did not test for implications of these findings to forensic identification methods.

There is a need to more thoroughly understand how minor deviations in radiographic orientation may affect forensic frontal sinus identifications, given the severe consequences of a mistaken identification or erroneous exclusion of identity. This study aims to assess how 5° vertical, horizontal, and diagonal (i.e., combined 5° vertical and horizontal) deviations in orientation from standard views affect frontal sinus shape as captured by outlines using a sample of computed tomography (CT) scans from 21 individuals. The increased sample size, inclusion of horizontal, vertical, and diagonal deviations, and testing of three standard radiographic orientations builds upon the previous literature, providing a more comprehensive study and the ability to directly compare results from the same sample across the orientations and deviations. This study also tests how these varying orientations may directly affect forensic identification, with a focus on the frontal sinus outline method devised by Christensen [15,16,28]. This method was chosen as it is one of the most cited methods for forensic sinus identification [30] and provides a means of capturing overall frontal sinus shape, which can then be quantitatively analyzed. The results of this study will help guide best practices in forensic frontal sinus identifications.

## 2. Materials and Methods

### 2.1. Materials

This study utilizes computed tomographic (CT) scans originating from the Robert J. Terry Anatomical Collection, National Museum of Natural History, Smithsonian Institution (Washington DC) [31]. The current sample included 21 adult crania (aged 20 to 95, average age = 51.667), with 13 African American (7 females, 6 males) and 8 European American (6 males, 2 females) individuals. The 21 crania were selected from a larger sample of CT scans publicly available from Lynn Copes’ website [32,33]. Only individuals possessing frontal sinuses above the supraorbital line, with no obvious signs of pathologies affecting the frontal sinus were utilized. Additional sample information and scanning protocols are provided by Copes [32,33]. Although the sample does not encompass broad ranges of temporal, population, or specific age variations, the crania included displayed a wide range of sinus size and morphology. Thus, this methodological study is able to assess effects of deviations on a broad range of individual sinus morphologies, as appropriate for our question. Although some studies have documented patterns in frontal sinus morphology between population, sex, or age groups or with variables such as body size and craniofacial morphology, these relationships are relatively weak [34,35,36], and the underlying factors contributing to such a high degree of frontal sinus variation, whether within or across groups, are unknown. These variations are described as differences in sinus appearance, size, and shape— all variables included in the present study. Thus, although this study does not include a highly diverse sample, the results of the analyses should be applicable across groups. 

For this study, the CT scans were imported into the program Amira5.6 [37], where semi-automatic processes were used to segment the frontal sinus, effectively creating a virtual endocast, and model the cranium following a previous study [38]. Both objects were digitally rendered and saved as two stereolithographic (.stl) models. Although there were two models, they maintained the same coordinate space and could be manipulated (e.g., oriented) together as if they were a single object. Care was taken not to employ smoothing techniques or any processing techniques that would alter frontal sinus morphology. 

### 2.2. Sinus Orientations

The aim of the study was to assess how minor deviations from common radiological views could affect the observed sinus morphology and how that may impact forensic identification methods. When deciding which radiological orientations to include in the study, common clinical and anthropological radiographic orientations (i.e., Frankfort Horizontal, PA-frontal, Caldwell’s, Water’s view) were considered. Specific definitions of these clinical views can vary by source, with some definitions focusing on soft tissue structures (e.g., nose against film) or the resultant radiographs (e.g., alignment of the petrous portions within the orbits), instead of specific osteometric landmarks. Clinical orientations for head radiography depend on both the positioning of the head relative to the X-ray film, as well as the angle of the beam trajectory. Given that this study utilizes CT scans to reduce subjectivity in positioning of these minor deviations in orientation, we could not emulate changes in beam trajectory (i.e., the CT scans are most similar to an X-ray beam trajectory perpendicular to the film); thus, this limitation was also considered when choosing which radiographic views to test. Priority was also given to orientations used in past frontal sinus and orientation studies for comparative purposes. 

**Figure 1:**
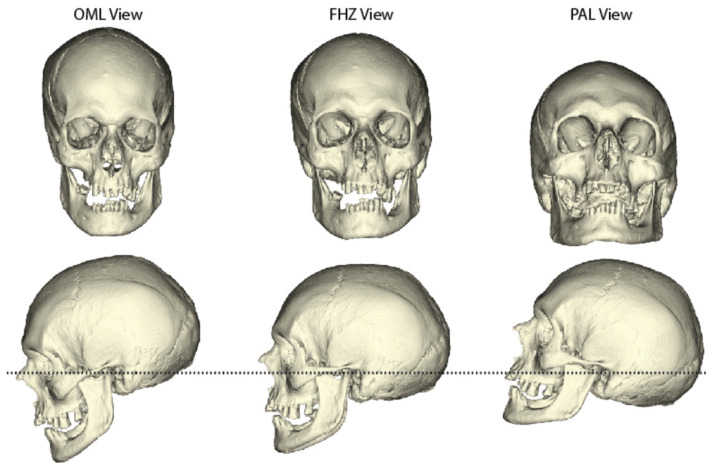
Standard cranial views utilized in this study from left to right: Orbitomeatal Line (OML), Frankfort Horizontal Plane (FHZ) and Porion-Alveolar Line (PAL). Dashed line represents the axial plane of orientation, see text for details.

Given the above considerations, three radiographic planes were chosen for evaluation: Frankfort Horizontal, Orbitomeatal, and Porion-Alveolar (see Figure 1). The Frankfort Horizontal Plane (FHZ) was chosen for several reasons: it is used in several clinical settings, particularly for occlusal and temporomandibular evaluations [39,40,41,42]; it is a common orientation in frontal sinus identification research [7,43,44,45]; it has been utilized in previous studies specifically investigating the role of varying orientations on frontal sinus morphology [25,26,27]; and it is most familiar to forensic anthropologists. For FHZ, crania are oriented such that the left-sided landmarks of *porion* (superior aspect of the external auditory meatus) and *orbitale* (most inferior margin of the orbit) are aligned in one axial plane, with left and right *porion* as level as possible (Figure 1, middle). For this study, FHZ was considered as the intermediate view, as the remaining views alter the crania inferiorly and superiorly relative to FHZ.

The Orbitomeatal Line (OML) was chosen as it is commonly referenced in clinical radiographic views, such as PA-frontal views, with X-ray beam trajectories following this axis and perpendicular to the film. Thus, not only is this view easy to replicate with CT scans, but antemortem radiographs obtained for forensic identification may frequently be in this position due to their use for evaluating the midfacial regions. For this view, crania are oriented such that the center of the external auditory meatus and the middle of the orbital cavity are aligned in the same axial plane, with left and right sides as level as possible. Note, Cruz and Gasperini [46] found that the OML is approximately 15° from FHZ, with the cranium rotated more inferiorly in OML.

The third view, which we termed the Porion-Alveolar Line (PAL), was primarily chosen for comparative purposes, as it represents a superior rotation of the head and is utilized in several previous studies investigating the effect of orientation on frontal sinus morphology. Following Silva et al. [25] and Nikolova et al. [26], we obtained this view by rotating the cranium 20° superiorly from FHZ (Figure 1, right). It is important to note, however, that these previous studies misleading refer to this orientation as “Caldwell view,” which does not match the clinical definition. Clinically, Caldwell’s view is obtained by having the patient put their forehead and nose against the X-ray film and then orienting the X-ray beam at a 15–20° angle to the film [24]. In this orientation, the head position resembles the OML view, but the X-ray beam traverses from the occipital bone (near *lambda*) through the mid-orbit region. The superior rotation of the cranium 20° from FHZ (as done by Silva et al. [25] and Nikolova et al. [26]), rotates the head in the opposite direction as the clinical Caldwell head orientation and results in an X-ray beam trajectory passing through the *porion* and the maxillary alveolus, very different from that of actual Caldwell’s view. Further, the end result of Caldwell’s view should consist of the petrous pyramids located in the lower third portion of the orbits [24], which is not evident in the figures provided by Nikolova et al. [26]. While we find this view informative for reasons below, we do not use the “Caldwell” notation in this study. Instead, we refer to it as the Porion-Alveolar Line (PAL), as this more accurately reflects the beam trajectory through the cranium. Despite not being defined as a typical clinical view, we include PAL here for comparative purposes given that these previous studies used this orientation, found that it was the most stable in terms of frontal sinus morphology across varying vertical orientations, and provided the clearest view of the sinus [25,26]. Its incorporation also provides additional insight into how superior vertical inclination of the cranium may affect frontal sinus morphology. Given the relationship of FHZ and OML axes, the PAL orientation can be inferred to be approximately 30° superiorly rotated from the OML line. While Water’s view, defined as 45° superior rotation from the OML view, is a clinical view that could have been investigated, it was not specifically tested in this study given that its extreme superior rotation has already been shown to be highly susceptible to vertical deviations [24,27,47]. Further, preliminary investigations in the current study indicated that Water’s view would result in several instances where the frontal sinus would be eliminated completely from view. The PAL view provides a test of a less-extreme vertical orientation.

### 2.3. Frontal Sinus Outlines

**Figure 2:**
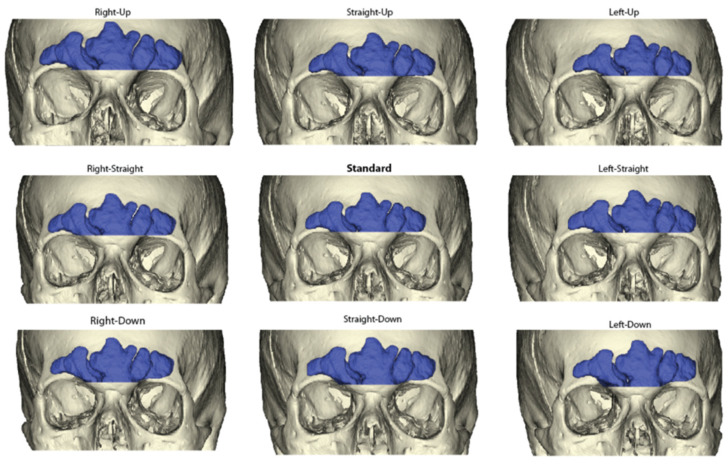
Graphical representation of the nine varying 5-degree orientations used in each view. Frankfurt Horizontal Plane pictured. Note actual 2D images used for analyses did not include the cranium.

The associated frontal sinus and cranial models for each individual were imported into the program 3DSlicer [48]. The models were oriented into these three main orientations (FHZ, OML, PAL) using cranial landmarks, and then eight additional views varying in 5° intervals from each of the main orientations, resulting in 27 radiographic views per individual. The Transformation module was used to digitally rotate the models to the defined degrees and alleviate human error in obtaining the 5° rotations. Within each view, the 5° deviations were defined from the standard orientation as horizontal deviations (5° left; 5° right), vertical deviations (5° up; 5° down), and diagonal deviations (5° left and up; 5° right and up; 5° left and down; 5° right and down). These are illustrated in Figure 2; note the cranial model is included for interpretative purposes only and was not included when obtaining sinus outlines as described below.

Following previous studies [15,16,23,28], the inferior border of the frontal sinus was demarcated at the level of the superior orbital margin in each view; the sinus remaining below the line was deleted from view using the Model Clipping tool in 3DSlicer. Once oriented correctly with the inferior border demarcated, the cranial model was hidden from the view, leaving the properly oriented and clipped sinus model above the superior orbital margin. The background was set as black, the sinus model set at white, and a 10cm scalebar was added. A two-dimensional (2D) image was then captured of the sinus and scalebar using the Annotations Screen Capture module in 3DSlicer. This resulted in a total of 567 images for 21 individuals. 

The 2D images were then imported into ImageJ [49] where they underwent further processing. First, image sizes were increased to 3000 pixels. Given that outline analyses require a single continuous outline, if the right and left lobes of the frontal sinus were separated (i.e., discontinuous) they were connected by a white line (set as 2 pixels thick). Additionally, each image was scaled according to the 10cm scale bar obtained from 3DSlicer, and frontal sinus area, maximum breadth (taken parallel to the supraorbital line), and maximum height (taken perpendicular to the supraorbital line) were collected using the Measurement tools in ImageJ. Maximum breadths and heights were taken twice by the same observer and then averaged.

Following Christensen [15,16,28], frontal sinus outlines were based on the external contour of the sinus, with the supraorbital line demarcating the inferior boundary. Elliptical Fourier analysis (EFA) was conducted on the outlines to quantitatively capture outline shape as a series of harmonics. Unlike other morphometric methods, EFA does not require homologous landmarks (which the frontal sinus lacks); instead, the outline shape is captured by harmonics, and resultant elliptical Fourier coefficients can be used to assess sinus shape (see [50] for a general review of EFA in forensic anthropology). EFA analyses were conducted using the SHAPE software [51] where the frontal sinus outlines were automatically digitized based on the 2D images of the white sinus models against the black background. The outlines were converted into numeric codes, referred to as “chain codes,” using the CHC module. Next, the CHC2NEF module was used to convert the codes into elliptical Fourier coefficients represented by 20 harmonics and normalized by the first harmonic. The resulting coefficients were then subjected to a principal component analysis (PCA) using the Princomp module, and the effective PCs (i.e., those with proportions larger than 1/*n*_coefficients_) were retained for all subsequent statistical analyses.

### 2.4. Statistical Analyses

Unless otherwise noted, all analyses were conducted in SPSS v28 [52], using a significance of 0.05. Initial exploration of the data indicated several measures violated the assumption of normality (i.e., Shapiro–Wilks *p*-values < 0.05). To be conservative, non-parametric statistics were utilized for all analyses. Spearman’s Rho correlation analyses were conducted to gain initial insights into how the PCs varied. To test for significant differences in sinus morphology due to varying orientations within each of the three views, Wilcoxon sign ranked tests were conducted. Owing to the assumption that investigators would place crania as close to the antemortem image as possible, and in order to reduce the number of pair-wise comparisons, we focused our analyses on the deviations from the standard orientation within each view, versus investigating orientations across the three major views. Specifically, the effects of the 5° variations on sinus variables (i.e., area, breadth, height, PCs) in the standard FHZ, OML, and PAL views were tested against their eight respective deviations in orientation. Due to multiple tests in this section, we applied a Bonferroni correction: significant differences were considered at the 0.006 alpha-level (0.05 divided by eight tests, per standard view). Descriptive statistics and plots were used to interpret differences and trends observed between the views and deviated orientations. Visualizations of the PCs and original sinus outlines were also utilized when interpreting how the orientations affected sinus shape.

The final portion of this study was to test the implication of varying orientation on frontal sinus identification. To accomplish this, multivariate Euclidean distances were calculated across the effective PCs between each varying orientation, both within individuals (intra-individual distances) and among different individuals (inter-individual distances). The assumption was that the intra-individual distances should be significantly less than the inter-individual distances. Three Euclidean distance matrices—one for each view— were created using the program PASSaGE2.0 [53]. Mann–Whitney U tests were conducted to statistically compare the pooled intra-individual distances (all orientations; *n* = 36 distances per individual, with 756 distances per view) to pooled inter-individual distances (all orientations; *n* = 1620 per individual, with a total of 34,020 distances per view). Using a one-tailed hypothesis, these analyses allowed us to directly test whether inter-individual distances were significantly greater than the intra-individual distances within each view. To test whether a specific view (FHZ, OML, or PAL) was less/more reliable than others, a Kruskal–Wallis analysis was conducted to test for significant differences in the intra-individual distances among the three views. If significant, follow-up Mann–Whitney U tests were conducted to directly test for specific differences among the three views. These analyses were conducted in SPSSv28 using a significance of 0.05, unless otherwise noted. 

Finally, to assess whether the slight deviations from standard orientations could affect forensic frontal sinus matches as based on Christensen’s outline method [15,54], we also took each outline (*n* = 189; 21 individuals and 9 outline views) and determined which outline they most closely matched to (i.e., least Euclidean distance). This was carried out within each of the standard views (i.e., all FHZ compared to all other FHZ outlines). If the deviations in orientations do not grossly affect positive identification, then the smallest distance (closest match) should be to an outline within the same individual and not an outline from a different individual.

## 3. Results

The PCA yielded eight effective PCs explaining a cumulative 92.57% of the variation. The results of Spearman’s Rho correlation analyses between the PCs and sinus variables are presented in Table 1. In terms of the sinus dimensions, PC1 most closely approximates sinus height, as indicated by the higher correlation coefficients, compared to PC2 and PC3. More specifically, PC1 (35.77% of the variation) largely tracked height and breadth dynamics; individuals with negative PC1 scores expressed superior inferiorly flatter sinuses (i.e., relatively larger breadth than height) compared to positive PC1 scores. PC2 (24.20%) appears to capture sinus complexity; outlines with more negative PC scores have several large “loops” and “indentations”, with some of the indentations approaching the supraorbital line, while those with more positive PC scores lack these indentations. PC3 (13.53%) tracks relative height changes in the outline, with negative PC3 scores representing sinuses with a distinctly higher midline (i.e., similar to a mountain peak) compared to lateral areas and positive PC3 scores representing sinuses with more equally distributed heights across the outline (i.e., similar to a plateau). The remaining PCs each explain less than 10% of the variation and were not univariately analyzed further. 

**Figure 3:**
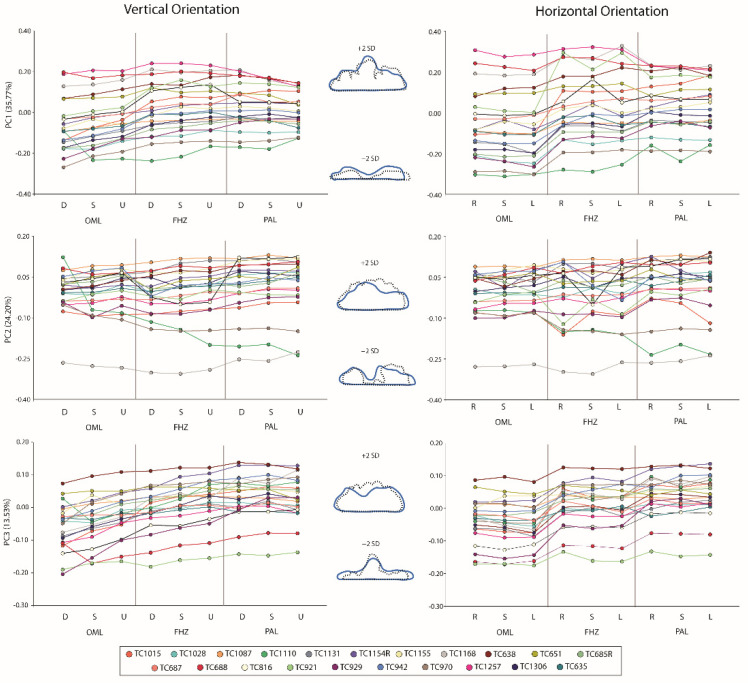
PC1 (top), PC2 (middle), and PC3 (bottom) values plotted against vertical orientations (left) and horizontal orientations (right) for each view; circles with same color scheme represent the same individual across all graphs (legend provided). PC contours also provided, with thick blue lines representing constructed +2 standard deviations (above) and −2 standard deviations (below) relative to the mean; black dashed lines representing actual outlines from an individual near the extremes of the axes. D, down; S, straight; U, up; R, right; L, left.

Table 2 provides descriptive statistics for PCs 1–3 in each orientation, along with the Wilcoxon sign ranked tests between the standard (i.e., non-deviated) view and each 5° variation of that view. PC1 and PC3 present with more significant differences compared to PC2, which likely relates to their relationship with size-related (i.e., relative height and breadth) shape changes. All significant differences occur within the FHZ and OML views and all significant differences involve some deviation in a vertical component (i.e., left-straight and right-straight deviations did not result in any significant differences). As individuals are oriented further inferiorly, they tend to display significantly shorter heights relative to breadth (i.e., more negative-loading PC1 and PC3s). This can be seen in Figure 3, which shows PC1–PC3 values (with associated contours) against 5° vertical and horizontal deviations (diagonal views not pictured).

**Figure 4:**
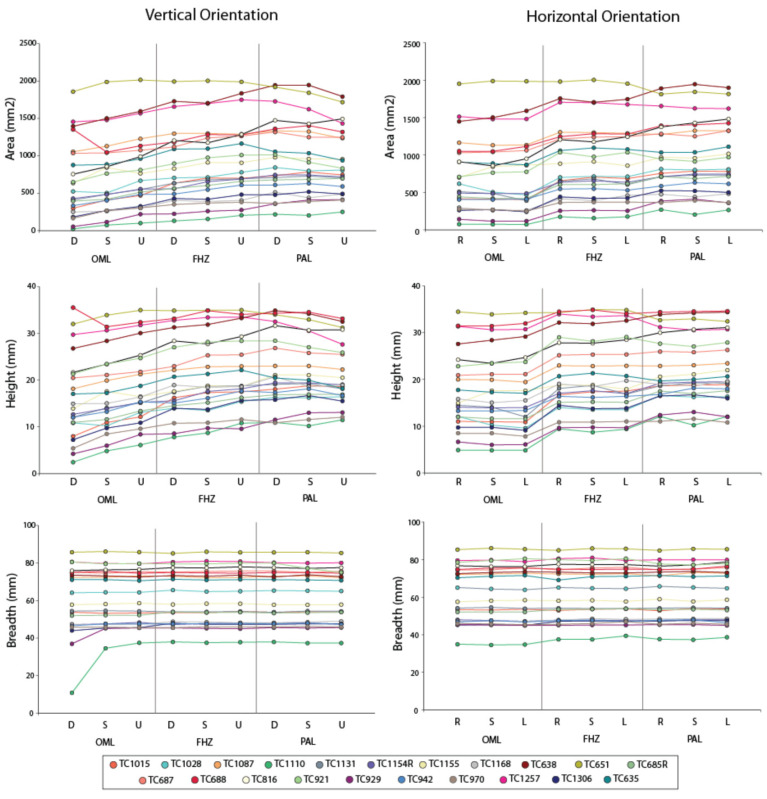
Sinus area (top), height (middle), and breadth (bottom) plotted against vertical orientations (left) and horizontal orientations (right) for each view: Orbitomeatal Line (OML), Frankfort Horizontal Plane (FHZ), and Porion-Alveolar Line (PAL). Circles with same color scheme represent the same individual across all graphs (legend provided). D, down; S, straight; U, up; R, right; L, left.

The interpretations of the PC results were confirmed by analyzing the sinus area, height, and breadth measurements. Table 3 provides descriptive statistics for these variables in each orientation, along with the Wilcoxon sign ranked tests between the standard and each varying 5° orientation within each view (e.g., FHZ standard vs. FHZ left up). OML and FHZ displayed significant differences in area and height for most views, while PAL only displayed significant differences in height. Again, deviations without any vertical component (i.e., right straight and left straight) did not result in significant differences. There were no significant differences in breadth among the 5° deviations and any of the three standard views. These results are best illustrated in Figure 4, which shows individual area (top graph), height (middle graph), and breadth (bottom graph) dimensions against 5° vertical and horizontal orientations (diagonal views not pictured). Note the relatively stable breadth dimensions, with one exception in the OML down view (see discussion).

### PC Distances 

**Figure 5:**
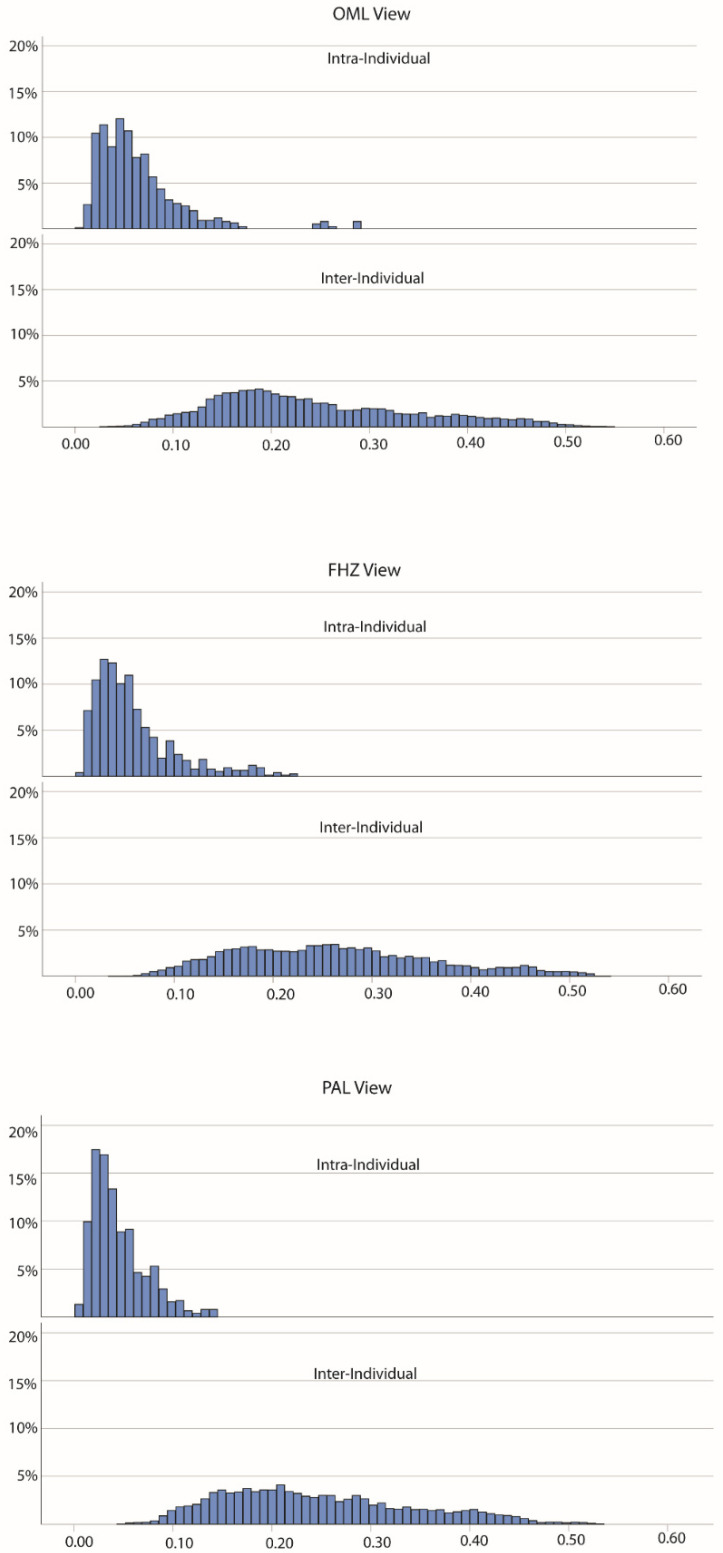
Percentage histogram of multivariate PC inter- and intra-individual distances for Orbitomeatal Line (OML; top); Frankfort Horizontal (FHZ; middle) and Porion-Alveolar Line (PAL; bottom) views.

**Figure 6:**
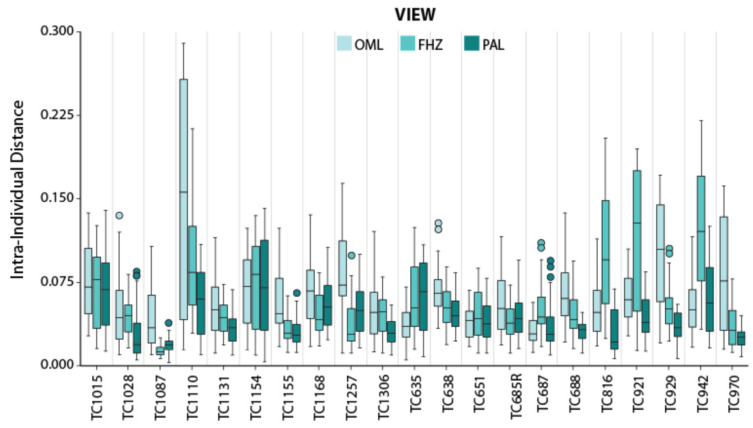
Box and whisker plots illustrating medians and quartiles for intra-individual distances for each individual in the three views: Orbitomeatal Line (OML), Frankfort Horizontal Plane (FHZ), and Porion-Alveolar Line (PAL).

Figure 5 provides histograms of the intra-individual and inter-individual distances for each standard view, while Table 4 provides the descriptive statistics and Mann–Whitney U Test results. For each of the three views, the intra-individual distances were significantly lower than the inter-individual differences (all *p*-values < 0.005). When comparing the intra-individual distances across the three views, a Kruskal–Wallis test indicated significant differences (test statistic = 118.22; *p* < 0.001). Follow up Mann–Whitney U tests on the intra-individual distances found that the significant differences between all views: OML versus FHZ (Z = −3.277; *p* = 0.001); OML versus PAL views (Z = −10.676; *p* < 0.001); and FHZ versus PAL views (Z = −7.252; *p* < 0.001). Figure 6 provides a boxplot for the intra-individual distances, by individual and view. Notably, the median and range of intra-individual distances are higher among OML views for most individuals compared to the FHZ and PAL views.

**Figure 7:**
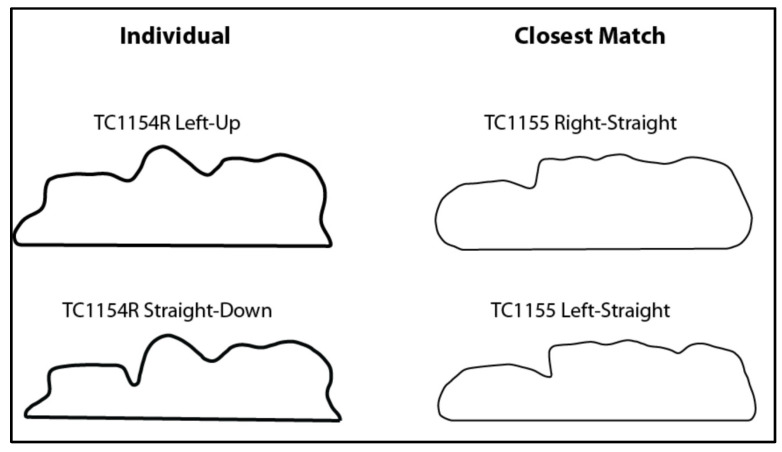
Outlines of the two mismatched individuals (TC1154, TC1155).

Still, all three views showed high reliability in outline matching. For the PAL and FHZ views, all 189 outlines matched with outlines from their same individual, for a correct match rate of 100%. The OML view had a correct match rate of 98.94%, with only two instances where outlines most closely matched to a different individual. Interestingly, both mismatched instances included the same two individuals, but different paired orientations: TC1154R left-up to TC1155 right-straight and TC1154R straight-down to TC1155 left-straight. Visual assessment of these outlines suggests striking morphological similarities, as seen in Figure 7. Overall, these results suggest that although the 5° deviations returned statistical differences in the Wilcoxon sign rank tests, these deviations are not likely to impact forensic sinus matches in practice, particularly for the PAL and FHZ views.

## 4. Discussion

Several studies suggest that when using the frontal sinus as an identification method, postmortem radiographs should be taken as closely as possible to the antemortem orientation [1,19,55,56]. However, a perfect alignment match can be challenging, if not impossible, and small deviations between the radiographic comparisons (e.g., 5°) is highly likely [29,57,58]. The current study investigated how small 5° deviations in vertical, horizontal, and diagonal axes may affect frontal sinus morphology within three clinically relevant views based on the Orbitomeatal Line (OML), Frankfort Horizontal Plane (FHZ), and the Porion-Alveolar Line (PAL). 

In terms of overall sinus dimensions, the current study found that sinus breadth remained relatively stable throughout the deviations, while sinus height was more affected by small variations in vertical orientation. This effect is illustrated in Figure 4 (middle left), which shows the progression of change in sinus height from the most inferiorly oriented view (OML straight-down) to the most superiorly oriented view (PAL straight-up). Changes affecting sinus height dimensions will also affect shape variables (PCs) related to sinus height–breadth dynamics (i.e., height relative to breadth). This effect was evident in the Wilcoxon sign rank tests, whereby deviations in vertical orientations resulted in significant differences in PC1 and PC3, both of which capture aspects of sinus height relative to breadth. As further support, horizontal changes without vertical re-alignment (e.g., simply looking left-straight or right-straight) did not present significant differences from the standard views. As discussed further below, these results are important for forensic frontal sinus matching methods that utilize measures of sinus height or variables associated with sinus height (e.g., area, height/breadth indices, or outlines). 

When comparing the three standard views, the PAL view showed to be the most stable, with the OML view the least stable, in terms of minor deviations altering sinus morphology. While the PAL view had significant differences in measured height with vertical deviations, all other measured and PC variables appear unaffected. Further evidence for reduced reliability in the OML view comes from the analyses on the Euclidean distances, which showed a higher range of intra-distance variation compared to the other views (Figure 5). As the OML view was the most inferiorly rotated of the three views investigated here, this suggests that more superiorly oriented radiological views are more stable in frontal sinus identifications. This is likely true up until a certain extent, as previous studies have shown that Water’s view (involving a superior rotation approximately 10°s higher than our PAL view) can be highly affected by orientation deviations and drastically affects the appearance of craniofacial structures [47]. In terms of morphological stability, frontal sinus dimensions seem to be more stable across varying orientations within more moderate views, such as the PAL and FHZ views utilized here. This is consistent with previous publications [25,26]. In fact, Nikolova et al. [26] found that sinus dimensions in their “Caldwell” view (the same view as our PAL view; see materials/methods) was the least affected by deviations in orientations.

However, previous studies also noted a more drastic alteration of sinus breadth versus height, which is contrary to the results found in the current study. Of several craniofacial measures, Riepert et al. [27] found frontal sinus breadth presented with some of the highest deviations with varying orientations. Nikolova et al. [26] found that any 5° inferior vertical orientations of the cranium from their standard “Caldwell”/PAL position resulted in a significant decrease in sinus breadth, while superior vertical orientations resulted in an increase in breadth. This likely relates to the fact that most sinuses are widest near their inferior base and the inferior border was determined at the superior orbital margin. As such, a superior tilting of the cranium would result in the base of the sinus being more prominent, while inferior tilting of the cranium would result in the base of the sinus dipping below the superior orbital line. Silva et al. [25] also found that 10° vertical deviations from their standard “Caldwell”/PAL position resulted in narrowing of breadths, likely due to the lateral edges of the sinus being lost from view in either direction. 

There could be several reasons why the current study did not also find significant differences in breadth. First, it should also be noted that the Silva et al. [25] study focused on relatively large degrees of variations (10°); there is a possibility that significant differences in sinus breadth would not have been found in smaller degrees of orientation, such as the 5°s measured here. Additionally, crania were manually repositioned to obtain each varying degree, which could have introduced an additional potential source of error [29]. As another consideration, both Silva et al. and Nikolova et al. utilized radiographic images, which incorporate measures of distortion inherent to radiographs, including issues of superimposition and magnification, that were not investigated here with CT-derived models. Yanagisaw and Smith [24] (p. 112) note that “the posterior tilt of the head… causes some distortion of the frontal sinuses because their vertical axes are not parallel to the film and the space between the frontal sinuses and the film is considerable.” It could very well be that these radiographic sources of visualization error more greatly affect sinus breadth versus height, which the current study did not capture (see Section 4.2) also see [27,58]. 

### 4.1. Effect on Sinus Identification

Still, despite significant differences between several orientations and views found in the current study, these deviations do not seem to largely affect potential identification as assessed by outline analyses (e.g., the Christensen method [15,24]). For all three views, Mann–Whitney U tests indicate that the intra-individual differences were significantly lower than the inter-individual differences. All three views also had high instances of true-positive matches, with OML at 98%, FHZ at 100%, and PAL at 100%. These results are similar to Christensen [15], who found that while there was some overlap where individual outlines most closely matched another individual, such occurrences were rare. Overall, this suggests that in most cases—regardless of varying 5° orientations or views—an individual’s outlines more closely resemble each other than outlines of other individuals. This is likely due to the already high inter-individualistic aspect of sinus morphology [27], which supersedes more subtle differences related to orientation. However, given that two outlines for the OML view did incorrectly match with another individual, caution is warranted when applying such quantitative methods of sinus identification. Although the outlines that erroneously matched in this study were strikingly similar (Figure 7), a simple visual assessment could likely distinguish the two. This suggests that current quantitative methods are not yet capable of distinguishing subtle differences or performing the complex interpretations undertaken by human observation. Thus, while there is a push to move towards more quantitative and objective methods in the forensic sciences (particularly in the U.S. since the Daubert guidelines [59,60] and 2009 National Academy of Sciences Report [61]), such methods may be more susceptible to noise and minor deviations. 

**Figure 8:**
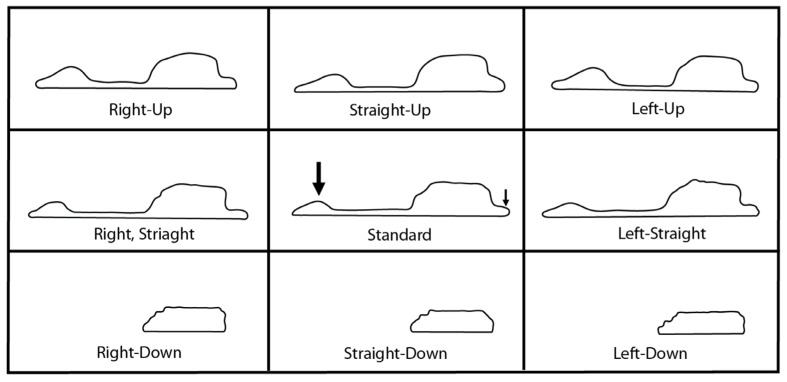
Example of individual (TC1110) outlines with relatively high intra-distances in the Orbitomeatal Line (OML) view (0.25–0.30) (see Figure 8 histogram); note the loss of the anatomical-right lobe and tail (large arrow), as well as the loss of the smaller anatomically left-sided arcade (small arrow), in the inferiorly oriented (Down) views. This drastically alters shape outline from two almost discontinuous lobes to a single plateau-like lobe. Outlines represent the normalized elliptical Fourier coefficients (based on 20-harmonics and aligned by the first harmonic).

Certain frontal sinus morphologies may also be more unique than others, and additional analyses are required to assess whether certain sinus variables (e.g., size, degree of complexity, etc.) are more prone to deviation issues or mismatches in larger and more diverse outline samples. There is already some indication that frontal sinus size affects the reliability of identification rates. As previously noted by Christensen [28] and Smith et al. [62], smaller sinuses are typically less complex (e.g., in terms of arcade number) and, thus, less diagnostic for identification purposes. Further, even slight vertical and/or horizontal variations could cause a smaller sinus to be partially, or even entirely, eliminated from view. Along these lines, the current study points to caution warranted when using identification methods on discontinuous sinuses and/or sinuses that have smaller lobes or lower arcades near the superior orbital border. In such cases, even small 5° variations may drastically alter the shape of the sinus. In the current study, this is best seen in Figure 4 (bottom, left), which shows the case of a single individual whose breadth was drastically smaller in the OML straight-down versus other views. Figure 8 investigates the OML outlines of this individual further. Note how, when in standard OML view, the individual outline presents with two distinct sinus lobes, with the anatomically right sinus being smaller (indicated by the large arrow) than the left; the left lobe also possesses a small arcade on its lateral edge (small arrow). As the individual is re-oriented inferiorly, the right lobe and smaller arcade completely disappear from view. This change would result in drastically different PC scores, particularly in terms of PC1 and PC3, which both track height-breadth dynamics. In a real-case scenario, if an investigator only had two images for this individual (e.g., straight-down and standard, see Figure 8), a true positive identification could be missed.

### 4.2. Limitations and Future Directions

While this study provides a preliminary understanding on how deviations in radiographic orientations can affect frontal sinus identification methods, there are several considerations for future studies. First, although we modeled this study based on the EFA method developed by Christensen [15,16,28,54], it was slightly modified from original analysis. Both studies utilized EFA, a standard method of comparing closed outlines, but the collection and analyses of these outlines differed. Using conventional radiographic images, Christensen manually traced outlines on acetate paper, then digitally converted the images into x, y coordinates. The act of manual tracing has potential for error due to differences in tracing the contours. The incorporation of 3D models here (see Materials and Methods) allowed the automatic capturing of outline shape without the potential effect (no matter how minimal) of manual tracing error. Further, unlike Christensen who utilized the x, y coefficients of the outline harmonics as their primary variable, we conducted a PCA to obtain PCs as our shape variables. The use of PCA has the advantage of simplifying the dataset, which are easier to analyze and interpret. In both approaches, the overall results were the same: the use of EFA on outline shape analyses provides a relatively robust method for frontal sinus outline identification, at least for larger continuous sinuses (see above). Still, additional studies further testing the inter- and intra-reliability of this method across a wide range of sinus sizes and observers is necessary to fully validate this method. Along these lines, additional studies focusing on relationships between frontal sinus patterns and body size and/or cranial–facial morphology would be beneficial. Indeed, while several studies attempt to discern such relationships across diverse human populations, such studies are conflicting and no consensus of the underlying factors explaining sinus morphology have been reached (for more discussion, see [34,36,38]). Further, the direct implications (if any) of these variations on frontal sinus morphology, particularly outline shape, to forensic identifications are lacking in the literature.

Of importance to consider here is that all frontal sinus identification methods, whether based on coding, metric, visualization, or outlines, take the same variables (e.g., height vs. breadth, arcade/scallop number and presentation, presence/absence of sinus lobes) into consideration when attempting to corroborate or negate a potential match. While we focused on a single outline method, the other methods could also be affected by the varying orientations altering sinus shape described here. For example, a coding method incorporating the number of arcades or presence/absence of sinus lobes (e.g., [7,22]) would understandably be affected if a lobe or arcade became unviewable with certain orientations. Additional testing across wider ranges of identification methods and among more diverse samples is needed to determine if any single method is more robust to alteration in orientations compared to others. 

Finally, the primary limitation of this study is that it does not directly emulate real-life scenarios, largely due to the incorporation of CT-derived models versus radiographs. While using 2D images of the segmented sinuses emulates the 2D view obtained in traditional radiographs, the CT-derived sinuses would not have been affected by radiographic-specific parameters, such as radiographic quality, magnification, distortion or human error in degree placement. Overall, CTs have several benefits over radiographs: they allow a clearer, 3D view of the sinuses, can be oriented in any direction, and digitally derived models can be modified to showcase soft tissue, if needed, or not [63]. Further, emerging technological advantages in the clinical sector will likely result in investigators being presented with more antemortem images from CT scans [64]. Owing to this, several studies attempt to create frontal sinus identification methods based on CT scans and digitally derived sinus models specifically [8,9,17,65,66,67,68,69,70,71,72]. However, in terms of postmortem images, all investigators may not have the time, resources, or experience to obtain and evaluate CT scans, let alone go through the process of creating frontal sinus models for identification purposes. Access to such technology may vary depending on geographic regions as well. While postmortem virtual autopsies utilizing CT scans (sometimes referred to as virtopsies or postmortem CTs) are relatively common among European countries [73], their incorporation in the United States is lagging. In fact, a relatively recent article from 2018 indicated that only four U.S. agencies have CT equipment available for regular use [74]. 

Although postmortem analog radiographs may still be more likely (at least in certain regions), we avoided two potential sources of error by utilizing digital frontal sinus models versus traditional radiographs in the current study. First, we more accurately and precisely positioned the cranium using digital means, avoiding error introduced by manual re-positioning crania on the X-ray tables [26,29]. Second, since the act of digitally or manually tracing outlines may impose additional error, we obtained 2D images of the models to automatically digitize frontal sinus outlines. Both steps allowed us to directly test the actual effect of varying orientation on this method, without additional sources of error and/or bias. However, this also means that the major sources of error introduced in varying orientations on frontal sinus morphology—issues of distortion, magnification, and superimposition of radiographs—could not be taken into consideration here. These effects were assessed by Nikolova et al. [26] who directly tested how changes in radiographic images taken from an industrial µCT scanner distorted actual linear measures. By directly comparing radiographic linear measures to a virtual frontal sinus endocast (i.e., model), they found breadth is more distorted than height dimensions. However, while they used an industrial µCT scanner, which has a fixed X-ray tube and flat panel detector similar to conventional radiography, it is unclear whether the beam passed through the crania from a posterior–anterior or anterior–posterior direction—an important distinction, as the differing orientation of the beam will pass through different layers of superimposed structures, varying the effects of distortion. In the clinical setting, the radiographic beams are typically aligned posteriorly–anteriorly through the cranium, which avoids direct radiation that could harm the patient’s orbital contents. But, there is further distortion of the sinus morphology as the beam travels through the present soft tissue, such as brain matter [75]. Future studies directly comparing frontal sinus identification methods between mixed modalities (e.g., CT scans vs. traditional radiographs) and with the presence/absence of soft tissue would be informative.

### 4.3. Recommendations on Sinus Identifications 

While there will hopefully be a move to include more advanced imagining technology at medicolegal agencies globally, we offer several recommendations when using the frontal sinus as an identification method with analog radiographs. Firstly, it is obvious that practitioners should aim to orient the cranium as close as possible to the antemortem comparative image. Besides the position of the skull, this may also involve matching the direction of X-ray (e.g., anterior–posterior or posterior–anterior) and the angle at which the X-ray enters the skull and encounters the film. For example, in true Caldwell position while the head is oriented horizontally to the film, the X-ray beam is oriented at a 15–20° angle. With this in mind, forensic anthropologists conducting radiographic comparisons for personal identification should ideally have training in radiographic techniques. An increase in collaborations between clinicians, radiographic technicians, and medicolegal practitioners can ensure a better understanding of radiological practices and consensus on terminology. 

In terms of application, the results presented here can help practitioners differentiate explainable from unexplainable differences when conducting radiographic comparisons of frontal sinus morphologies. Although this study focused on one sinus outline method of identification, understanding the sinus shape variations that are expected with slight orientation differences can be used to better interpret the results of other sinus identification methods. Differences in vertical orientation of the crania can be expected to affect sinus height and/or the presence of smaller lobes/arcades, particularly those near the supraorbital border. In such cases, identification methods that rely on height measurements or counts of lobes/arcades should be avoided. If applied, a visual comparison should be used to confirm results, taking into account these expected changes with orientation, to ensure that a correct identity is not erroneously excluded due to these methodological errors. Keep in mind that such variation may be more drastic in more extreme views, such as Water’s view (common in clinical settings) and/or the OML view assessed here. Ultimately, although there is a push in forensics towards more quantitative methods such as EFA/outline analyses, coding methods, and metric analyses for the frontal sinus, these more objective methods may be more sensitive to slight deviations in the capture of the radiographic frontal sinus. Until more robust methods of quantitatively describing the frontal sinus morphology are developed or the use of CT technology becomes more commonplace across medicolegal agencies globally, visual comparisons, albeit more subjective, are likely more capable of interpreting such explainable differences between radiographs.

## 5. Conclusions

Overall, the current study found that the EFA outline method for identification developed by Christensen [15,16,28] is relatively robust to small 5° variations in orientation. However, in conjunction with previous studies, it is evident that reliability of frontal sinus identification methods is largely contingent on the view being imitated, the directionality of the deviation, and actual sinus morphology. Furthermore, based on our results, Christensen’s EFA outline method would appear to be most reliable on larger sinuses, particularly those that are superior inferiorly tall and medio-laterally wide without discontinuous lobes (i.e., the right and left sinuses are touching), although additional testing is needed to validate this method. For any method (e.g., outline, coding, and linear), caution is warranted when attempting to identify individuals with small sinuses, particularly sinuses possessing smaller discontinuous lobes. 

In terms of clinical views, small degrees of varying orientation within an intermediate range of standard clinical views (e.g., FHZ, PAL, and true Caldwell view) would be more reliable compared to other views. Caution is highly warranted if attempting to match antemortem radiographs taken in more extreme vertical orientations, including those based on the Orbitomeatal Line (e.g., posterior–anterior frontal view) and/or or Water’s view, as small deviations from that standard view can have more drastic effects on the presentation of sinus morphology. Finally, the results of this CT-based study should be considered limited, as additional sources of distortion common in traditional radiographs (e.g., angulation, magnification, presence of soft tissue, and superimposition) will likely have greater alterations to sinus morphology, particularly breadth, than that presented here. The results of this study assist practitioners in better understanding and interpreting explainable and unexplainable differences between radiographs.

## Figures and Tables

**Table 1:** Results of Spearman’s Rho correlation analyses (with correlation coefficients and *p*-values) between sinus variables and the principle components (PCs) representing >10% of variation.

	Area	Breadth	Height	PC1	PC2	PC3
Area	-	0.900	0.921	0.666	0.465	0.067
*p*-value	-	<0.001 *	<0.001 *	<0.001 *	<0.001 *	0.111
Breadth	0.900	-	0.873	0.615	0.295	−0.249
*p*-value	<0.001 *	-	<0.001 *	<0.001 *	<0.001 *	<0.001 *
Height	0.931	0.873	-	0.840	0.380	0.019
*p*-value	<0.001 *	<0.001 *	-	<0.001*	<0.001 *	0.658
PC1	0.666	0.615	0.840	-	0.092	0.068
*p*-value	<0.001 *	<0.001 *	<0.001 *	-	0.029 *	0.104
PC2	0.465	0.295	0.380	0.092	-	0.109
*p*-value	<0.001 *	<0.001 *	<0.001 *	0.029 *	-	0.009 *
PC3	0.067	−0.249	0.019	0.0368	0.109	-
*p*-value	0.111	<0.001 *	0.658	0.104	0.009 *	-

* Bold text with asterisk indicates significance at the alpha level of 0.05.

**Table 2:** Median and inter-quartile range (IQR) statistics for PC1–PC3 across the three views and varying orientation. Wilcoxon sign rank tests (Z scores and significance) also provided for each orientation versus respective standard view.

Orientation	PC1		PC2		PC3	
	Median (IQR)	Z	Median (IQR)	Z	Median (IQR)	Z
OML Standard	−0.075 (0.187)	−	0.009 (0.109)	−	−0.044 (0.094)	−
Straight Down	−0.090 (0.184)	−2.798 *	0.005 (0.082)	−0.261	−0.042 (0.097)	−2.450
Straight Up	−0.055 (0.174)	−3.424 *	0.022 (0.109)	−3.076 *	−0.018 (0.069)	−3.389 *
Right Straight	−0.079 (0.191)	−1.477	−0.004 (0.128)	−2.138	−0.046 (0.090)	−1.373
Right Down	−0.058 (0.171)	−2.798 *	0.040 (0.144)	−1.651	−0.015 (0.065)	−2.768 *
Right Up	−0.065 (0.177)	−3.076 *	0.004 (0.071)	−0.052	−0.052 (0.076)	−2.763 *
Left Straight	−0.068 (0.185)	−1.408	0.005 (0.123)	−0.226	−0.030 (0.077)	−2.450
Left Down	−0.065 (0.187)	−2.555	0.017 (0.133)	−1.651	−0.009 (0.061)	−3.250 *
Left Up	−0.099 (0.163)	−3.111 *	0.008 (0.095)	−0.087	−0.047 (0.072)	−1.929
FHZ Standard	0.027 (0.188)	−	0.012 (0.119)	−	0.025 (0.066)	−
Straight Down	−0.009 (0.193)	−2.798 *	−0.005 (0.118)	−1.547	−0.001 (0.058)	−3.667 *
Straight Up	0.018 (0.180)	−1.964	0.019 (0.115)	−2.868 *	0.034 (0.070)	−3.215 *
Right Straight	−0.013 (0.206)	−0.226	0.021 (0.167)	−0.365	0.028 (0.069)	−0.921
Right Down	−0.031 (0.204)	−2.728 *	−0.011 (0.152)	−0.261	−0.004 (0.075)	−3.806 *
Right Up	0.004 (0.179)	−0.904	0.027 (0.127)	−2.763 *	0.027 (0.066)	−2.589
Left Straight	−0.016 (0.175)	−0.956	0.026 (0.179)	−0.608	0.025 (0.073)	−1.477
Left Down	−0.009 (0.188)	−3.041 *	0.013 (0.171)	−0.504	0.018 (0.070)	−2.311
Left Up	0.007 (0.195)	−0.991	0.028 (0.173)	−1.130	0.033 (0.075)	**−3.945** *
PAL Standard	0.021 (0.163)	−	0.042 (0.118)	−	0.042 (0.062)	−
Straight Down	0.029 (0.155)	−1.894	0.028 (0.120)	−2.346	0.032 (0.079)	−1.130
Straight Up	0.006 (0.163)	−2.207	0.054 (0.121)	−0.365	0.027 (0.087)	−1.790
Right Straight	0.037 (0.177)	−0.365	0.040 (0.132)	−0.365	0.034 (0.071)	−1.095
Right Down	0.030 (0.164)	−2.103	0.030 (0.108)	−2.103	0.034 (0.077)	−0.295
Right Up	0.007 (0.165)	−1.721	0.042 (0.116)	−0.400	0.026 (0.089)	−0.504
Left Straight	0.014 (0.147)	−0.156	0.048 (0.119)	−0.261	0.040 (0.066)	−0.261
Left Down	0.023 (0.162)	−1.581	0.045 (0.133)	−0.956	0.041 (0.079)	−0.017
Left Up	−0.004 (0.162)	−2.207	0.055 (0.126)	−1.130	0.026 (0.087)	−0.747

* Bold text with asterisk indicates significance at the Bonferroni-adjusted alpha level of 0.006.

**Table 3:** Median and inter-quartile range (IQR) statistics for sinus variables across the three views (Orbitomeatal Line, OML; Frankfort Horizontal Plane, FHZ; and Porion-Alveolar Line, PAL) and varying orientations. Wilcoxon sign rank tests (Z scores and significance) also provided for each orientation versus respective standard view.

Orientation	Area (cm^2^)		Height (cm)		Breadth (cm)	
	Median (IQR)	Z	Median (IQR)	Z	Median (IQR)	Z
OML Standard	5.037 (7.077)	−	1.505 (1.287)	−	**5.815 (2.769)**	−
Straight Down	5.251 (7.719)	−3.146 *	1.394 (1.209)	−3.181 *	5.783 (2.823)	−1.303
Straight Up	6.681 (7.064)	−3.597 *	1.638 (1.239)	−3.806 *	5.862 (2.753)	−0.800
Right Straight	4.867 (7.640)	−0.382	1.553 (1.399)	−0.226	5.846 (2.866)	−0.678
Right Down	5.458 (6.929)	−2.728 *	1.567 (1.316)	−3.163 *	5.780 (2.785)	−0.608
Right Up	5.222 (6.370)	−3.563 *	1.385 (1.247)	−3.667 *	5.859 (2.864)	−0.417
Left Straight	6.160 (6.903)	−0.896	1.496 (1.204)	−1.717	5.757 (2.754)	−1.304
Left Down	6.332 (7.014)	−3.041 *	1.678 (1.356)	−3.250 *	5.763 (2.698)	−1.512
Left Up	5.450 (6.715)	−3.563 *	1.451 (1.235)	−3.389 *	5.801 (2.757)	−1.981
FHZ Standard	7.129 (7.775)	−	1.875 (1.347)	−	5.838 (2.742)	−
Straight Down	7.063 (7.337)	−3.007 *	1.762 (1.342)	−3.233 *	5.793 (2.731)	−0.463
Straight Up	7.806 (7.385)	−3.493 *	1.866 (1.292)	−3.007 *	5.836 (2.734)	−1.565
Right Straight	7.118 (7.689)	−0.504	1.793 (1.428)	−0.574	5.770 (2.807)	−0.205
Right Down	6.956 (7.918)	−3.389 *	1.739 (1.386)	−4.015 *	5.788 (2.779)	−0.017
Right Up	7.367 (7.826)	−3.424 *	2.019 (1.262)	−2.798 *	5.836 (2.859)	−1.026
Left Straight	7.015 (7.363)	−0.678	1.879 (1.366)	−0.330	5.814 (2.709)	−1.321
Left Down	6.534 (7.482)	−3.736 *	1.738 (1.391)	−3.910 *	5.788 (2.743)	−2.312
Left Up	7.525 (7.555)	−2.485	1.965 (1.298)	−3.245 *	2.709 (2.753)	−1.651
PAL Standard	8.002 (7.883)	−	1.945 (1.196)	−	5.775 (2.708)	−
Straight Down	8.446 (7.853)	−1.095	2.050 (1.348)	−3.233 *	5.780 (2.753)	−0.417
Straight Up	8.084 (7.420)	−2.033	1.897 (2.169)	−3.007 *	5.790 (2.729)	−1.363
Right Straight	8.081 (8.182)	−1.303	1.958 (1.237)	−0.574	5.869 (2.840)	−0.037
Right Down	7.999 (7.664)	−0.226	2.041 (1.249)	−4.015 *	5.854 (2.765)	−0.672
Right Up	7.948 (7.542)	−1.721	1.932 (1.093)	−2.798 *	5.812 (2.799)	−0.485
Left Straight	8.119 (7.747)	−0.817	1.920 (1.214)	−0.330	5.897 (2.710)	−1.547
Left Down	7.730 (16.219)	−0.261	2.028 (1.303)	−3.910 *	5.875 (2.730)	−1.095
Left Up	7.741 (7.408)	−2.172	1.930 (1.140)	−3.245 *	5.770 (2.657)	−0.672

* Bold text with asterisk indicates significance at the Bonferroni-adjusted alpha level of 0.006.

**Table 4:** Median and interquartile range (IQR) values, with Mann–Whitney U results for the multivariate PC intra- and inter-individual distances across the three views: Orbitomeatal Line (OML), Frankfort Horizontal Plane (FHZ), and Porion-Alveolar Line (PAL).

View	Intra-Distances	Inter-Distances	Z
	Median (IQR)	Median (IQR)	
OML	0.053 (0.045)	0.222 (0.144)	−43.681 *
FHZ	0.048 (0.043)	0.253 (0.145)	−44.868 *
PAL	0.035 (0.033)	0.225 (0.136)	−46.183 *

* Bold text with asterisk indicates significance at the 0.05 level.

## Data Availability

CT scans publicly available online. Other data available on request from the authors.

## Abbreviations

CT	computed tomographic
EFA	elliptical Fourier analysis
FHZ	Frankfort Horizontal Plane
OML	Orbitomeatal Line
PA	posterior–anterior
PAL	Porion-Alveolar Line
PCA	principal components analysis
TC	Terry Collection
